# Impact of Longkong Pericarp Extract on the Physicochemical Properties of Alginate-Based Edible Nanoparticle Coatings and Quality Maintenance of Shrimp (*Penaeus monodon*) during Refrigerated Storage

**DOI:** 10.3390/foods12051103

**Published:** 2023-03-05

**Authors:** Narin Charoenphun, Bharathipriya Rajasekaran, Suguna Palanisamy, Karthikeyan Venkatachalam

**Affiliations:** 1 Faculty of Science and Arts, Burapha University, Chanthaburi Campus, Chanthaburi 22170, Thailand; 2 International Center of Excellence in Seafood Science and Innovation, Faculty of Agro-Industry, Prince of Songkla University, Songkhla 90110, Thailand; 3 Faculty of Innovative Agriculture and Fishery Establishment Project, Prince of Songkla University, Surat Thani Campus, Surat Thani 84000, Thailand

**Keywords:** edible coating, antimicrobial activity, antioxidant activity, shelf-life extension, quality attributes

## Abstract

The objective of this study was to evaluate the impact of varying concentrations of longkong pericarp extract (LPE) on the physicochemical properties of alginate-based edible nanoparticle coatings (NP-ALG) on shrimp. For developing the nanoparticles, the alginate coating emulsion with different LPE concentrations (0.5, 1.0, and 1.5%) was ultrasonicated at 210 W with a frequency of 20 kHz for 10 min and a pulse duration of 1s on and 4 off. After that, the coating emulsion was separated into four treatments (T): T1: Coating solution containing basic ALG composition and without the addition of LPE or ultrasonication treatment; T2: ALG coating solution converted into nano-sized particles with ultrasonication and containing 0.5% LPE; T3: ALG coating solution converted into nano-sized particles with ultrasonication and containing 1.0% LPE; T4: ALG coating solution converted into nano-sized particles with ultrasonication and containing 1.5% LPE. A control (C) was also used, where distilled water was used instead of ALG coating. Before coating the shrimp, all the coating materials were tested for pH, viscosity, turbidity, whiteness index, particle size, and polydispersity index. The control samples had the highest pH and whiteness index and was followed by the lowest viscosity and turbidity (*p* < 0.05). Among the T1–T4 coating materials, T4 coating had higher turbidity, particle size, polydispersity index, but lower pH, viscosity, and whiteness index (*p* < 0.05). To study the quality and shelf-life of the shrimp, all coated shrimp samples were refrigerated at 4 °C for a period of 14 days. At 2-day intervals, physiochemical and microbial analyses were performed. The coated shrimp also had a lower increase in pH and weight loss over the storage period (*p* < 0.05). Coatings containing 1.5% LPE significantly reduced the polyphenol oxidase activity in the shrimp (*p* > 0.05). The addition of LPE to NP-ALG coatings demonstrated dose-dependent antioxidant activity against protein and lipid oxidation. The highest LPE concentration (1.5%) led to increased total and reactive sulfhydryl content, along with a significant decrease in carbonyl content, peroxide value, thiobarbituric acid reactive substances, p-anisidine, and totox values at the end of the storage period (*p* < 0.05). Additionally, NP-ALG-LPE coated shrimp samples exhibited an excellent antimicrobial property and significantly inhibited the growth of total viable count, lactic acid bacteria, Enterobacteriaceae, and psychotropic bacteria during storage. These results suggested that NP-ALG-LPE 1.5% coatings effectively maintained the quality as well as extended the shelf-life of shrimp during 14 days of refrigerated storage. Therefore, the use of nanoparticle-based LPE edible coating could be a new and effective way to maintain the quality of shrimp during prolonged storage.

## 1. Introduction

Shrimp has become increasingly popular among consumers for its distinctive taste, but it is not just a delicacy. It is also a rich source of protein, healthy PUFA, vitamins, and minerals [1]. The high moisture content and various nutrients in shrimp makes it more vulnerable to changes in physical, biochemical, and microbiological characteristics and such alterations can negatively impact the quality and shelf-life of shrimp, ultimately resulting in reduced market value [2]. Several chemicals including ethylenediaminetetraacetic acid, benzoic acid, polyphosphates, ascorbic acid, and sodium chloride have been tried as preservatives to maintain the quality and extend the shelf-life of shrimp during storage. However, sulfite-based formulation produces allergic reactions, thus synthetic preservatives exert an adverse effect on human health. Moreover, to preserve the quality of shrimp during prolonged storage, various advanced techniques have been studied, such as modified atmospheric packaging, vacuum packaging, high-pressure treatment, the use of plant extract, and applying edible coatings [3]. The edible coating is a promising and reliable method for extending the shelf-life of shrimp among the various techniques studied [4]. It creates a barrier against oxygen, slows down oxidation, prevents microbial contamination, reduces moisture loss, and preserves flavor [5]. Edible coatings also serve as a means of delivering food additives, such as antioxidants and antimicrobial agents. Using natural plant extracts in these coatings can extend the shelf-life of perishable foods, such as fruits, vegetables, and seafood [6]. Alginate (ALG), a polysaccharide derived from brown algae, is widely used as a coating material. It is considered a GRAS (generally recognized as safe) substance and is composed of D-mannuronic acid and L-guluronic acid. Alginate is popular as a coating material because it can form a strong gel and maintain its insolubility when reacting with multivalent cations [7]. Many studies have shown that incorporating preservatives into coatings can help prevent quality changes in perishable foods during prolonged storage [1,8]. However, consumers often prefer preservatives that come from natural sources to ensure the safety of their food. Phenolic compounds, which are widely found in plants, are one such example of these natural preservatives. Phenolic compounds are well known for their antimicrobial and antioxidant properties, making them a potent alternative to synthetic agents [9]. Longkong (*Aglaia dookkoo Griff.*) is an economically valuable, non-climacteric tropical fruit belonging to the Meliaceae family, primarily found in southern Thailand [4]. Longkong fruit is composed of three main parts—pericarp, flesh, and seeds—and the pericarp contains a high level of polyphenols. Studies indicate that longkong pericarp extract (LPE) exhibits multiple biological and pharmacological effects, including radical scavenging, germicidal, cytostatic, antimalarial, and depigmentation [10]. In addition, LPE contains a rich source of lansic acid, lansiosides, lansiolic acid, and iso-onoceratriene. These chemical compounds in LPE could control the hormonal imbalance in humans and promote anti-baldness, antipyretic and anti-feeding activities. Nevertheless, ellagic acid and corilagin in the LPE could promote anti-fibrosis and anti-glaucoma effects. Incorporating nanotechnology and natural plant extracts into the coating medium is an effective approach to preserve the stability of bioactive compounds and as well as food products against deterioration from oxidation and microorganism [11]. Additionally, the use of nanotechnology to produce particles of nano-dimension increases the surface area per unit weight, which results in better dispersion of the active substances in the coating medium, thereby enhancing its functionality and bioactivity [12]. Furthermore, the use of nano-sized particles allows for the controlled and gradual release of bioactive substances during prolonged storage [13]. Additionally, nanotechnology can incorporate active substances without altering the sensory characteristics of foods and increase their shelf-life [14]. Although, the antibacterial activity of LPE and alginate coating is well known, their combination, especially in the nanoparticles system, has not been studied. Therefore, the present study utilized this opportunity to examine the properties of LPE-added ALG-based edible nanoparticles coating and to investigate their preventive effect on the quality maintenance of shrimp during 14 days of storage at refrigerated conditions.

## 2. Materials and Methods

### 2.1. Raw Materials, Chemicals, and Reagents

Longkong fruits fully ripe were harvested from a local garden in Surat Thani province, Thailand. Black tiger shrimp (*Penaeus monodon*) measuring 6–8 cm in length were purchased from a nearby farm. The shrimp were placed on ice at a ratio of 1:2 (shrimp:ice) and transported to the lab within an hour. Upon arrival, the shrimp were rinsed with cold water and kept on ice until use, not exceeding 5 h. Food-grade sodium alginate (Keltone LV, ISP, San Diego, CA, USA) was used as a biopolymer for coating formulation. The analytical-grade solvents and chemical agents utilized in this study encompassed chloroform, ethanol, methanol, ethyl acetate, sodium hydroxide, Triton X-100, sodium chloride, 1-3,4-dihydroxyphenylalanine (DOPA), Tris, glycine, sodium ammonium sulfate, 5,5′-dithio-bis-(2-nitrobenzoic acid) (DNTB), ethylenediamine tetra acetic acid (EDTA), guanidine chlorate, dipotassium hydrogen phosphate, sulfosalicylic acid, potassium dihydrogen phosphate, hydrochloric acid, potassium iodide, sodium thiosulfate, urea, anhydrous sodium sulfate, trichloroacetic acid (TCA), ascorbic acid, thiobarbituric acid (TBA) (Merck, Darmstadt, Germany), Tween 80 (Labchem, Zelienople, PA, USA), 1,1,3,3-tetramethoxypropane (MDA) (Sigma-Aldrich, St. Louis, MO, USA), and acetic acid (Lab-Scan, Pathum Wan, Bangkok, Thailand). The media used for the microbiological analyses, namely plate count agar, peptone, deMan, Rogosa, Sharpe (MRS) agar, and violet red bile glucose agar (VRBG), were all analytical grade and purchased from Merck, Darmstadt, Germany.

### 2.2. Preparation of Longkong Pericarp Extract (LPE)

Upon the arrival of the longkong fruits, their pericarps were isolated from the flesh and washed using cold water with 2% ascorbic acid. The longkong pericarp extract (LPE) was obtained as guided by Nagarajan et al. [4] with some modifications. First, the pericarps were dried in a hot air oven at 40 °C until a consistent weight was reached, and the dried sample was grounded into fine powder. To prepare the LPE, 5 g of pericarp powder was mixed with 100 mL of absolute ethanol. The mixture was then heated with agitation in a water bath at 40 °C for 4 h. The resulting solution was then placed in the solvent evaporator to remove the solvent thoroughly at 40 °C. The final LPE was freeze-dried, stored in a sealed amber bottle, and kept at −20 °C until required for further experimentation.

### 2.3. Preparation of Coating Solution

The ALG coating solution was prepared by following the method of Sharifimehr et al. [5] with some modifications, dissolving 1% ALG (*w*/*v*) and 4% Tween 80 (*v*/*v*) in distilled water, and then adding LPE at different concentrations while constantly stirring. The mixture was thoroughly stirred using a magnetic stirrer to obtain a homogenous solution. The particle size of the coating was reduced to the nanoscale using ultrasonication (Hielscher UP200Ht, Hielscher Ultrasonics GmbH, Teltow, Germany) at 210 W, a frequency of 20 kHz for 10 min with a pulse duration of 1 s on and 4 s off. The temperature was maintained at 25 °C during the ultrasonication process. Four treatments (T) were used: T1: Coating solution containing basic ALG composition and without the addition of LPE or ultrasonication treatment; T2: ALG coating solution converted into nano-sized particles with ultrasonication and containing 0.5% LPE; T3: ALG coating solution converted into nano-sized particles with ultrasonication and containing 1.0% LPE; T4: ALG coating solution converted into nano-sized particles with ultrasonication and containing 1.5% LPE. A control (C) was also used, where distilled water was used instead of ALG coating. All the tested coating solutions were measured for physicochemical properties as shown in Section 2.5.

### 2.4. Physicochemical Analysis of Coating Solutions

#### 2.4.1. pH

The pH of the coating solution was measured using a digital pH meter (Mettler-Toledo GmbH, Giessen, Germany).

#### 2.4.2. Viscosity

To determine the viscosity of the coating solution, a digital tabletop Brookfield viscometer (Brookfield DVE viscometer, Middleborough, MA, USA) was utilized. First, 150 mL of the coating solution was placed in a beaker measuring 70 mm in diameter and 125 mm in height. A Viscometer equipped with a number 2 spindle, set to run at a speed of 12 rpm, was used to measure viscosity. The outcomes were expressed in centipoise (cP).

#### 2.4.3. Turbidity

The turbidity of the coating solution was measured using a turbidimeter (Hanna Instruments, model HI 93703, Woonsocket, RI, USA), and the results were expressed in percentages.

#### 2.4.4. Whiteness Index

The HunterLab colorimeter (MiniScan EZ 4000, Hunter Associates, Inc., Reston, VA, USA) was utilized to determine the color of the coating solution following the methodology outlined by Josewin et al. [15]. After calibrating the instrument using a white standard plate (*L* = 91.83, *a* = −0.73, *b* = 1.52), the lightness (L*), redness/greenness (a*), and yellowness/blueness (b*) were measured. The whiteness index (WI) was subsequently calculated using the following formula:

(1) $$\mathrm{WI}=100-\sqrt{{(100-L*)}^{2}{+(a*)}^{2}}{+(b*)}^{2}$$

#### 2.4.5. Particle Size and Polydispersity Index (PDI)

The particle size and polydispersity index (PDI) were assessed using a method adapted from Venkatachalam [16]. Backscatter detection at a 170° scattering angle was utilized in the process. The sample was stabilized within the device for 60 s before data collection at 25 °C. The particle size outcomes were reported in nanometers, while the PDI values were expressed as the polydispersity index.

### 2.5. Coating and Storage of Shrimps

**Figure 1:**
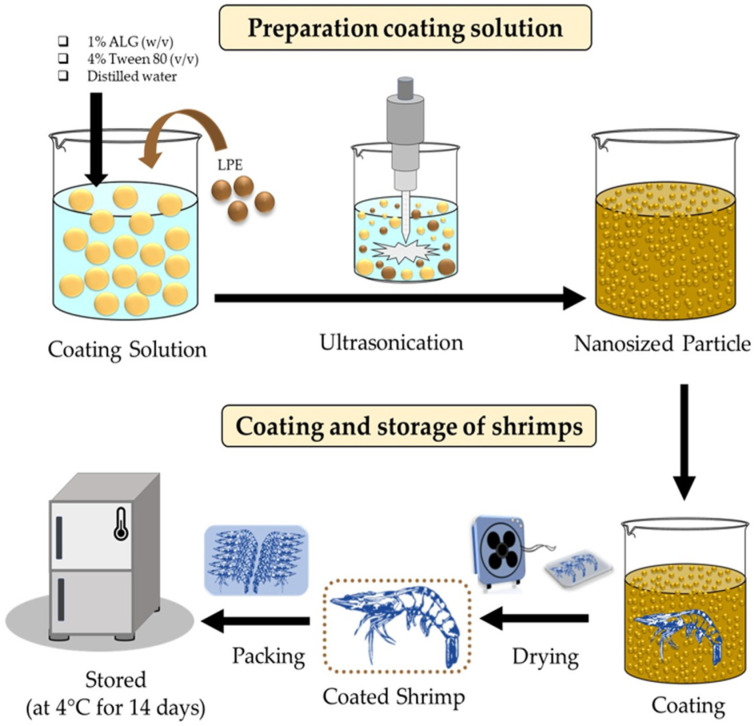
Schematic illustration of nanoparticle formation using ultrasonication and coating of shrimp using the LPE-added alginate-based nanoparticle edible coating.

The schematic illustration of the ALG coating preparation and coating of the shrimp is shown in Figure 1. The shrimp at a refrigerated temperature (4–8 °C) were coated by immersing them fully in a respective coating solution, as described in Section 2.4, and subsequently, the coated shrimps were placed on a wire rack for 1 min to allow excess coating material to drip off. The drying process of the coating material on the surface of the shrimp was facilitated by exposing coated shrimps to air blown by an electric fan. Next, the shrimps were arranged in polystyrene trays with 12 shrimps per tray, then wrapped with polyolefin film, and kept at 4 °C for 14 days. At every two-day interval during the storage period, samples were chosen randomly for shelf-life analysis as per the protocol described in Section 2.6.

### 2.6. Shelf-Life Analyses of NP-ALG-LPE Coated Shrimp during Storage

#### 2.6.1. Weight Loss

Prior to weighing, the stored shrimps were first dried off from any surface moisture by using a clean paper towel. Then, they weighed on an electronic weighing balance (SECURA124-1CIT, Sartorius, Goettingen, Germany). The following formula was used to determine the weight loss (WL) in the samples [17]:

(2) $$\mathrm{WL}\;(\%)=\frac{W_{1}-W_{2}}{W_{1}}\times 100$$

where W_1_ and W_2_ denote the weight of shrimp on the initial and final day of storage, respectively.

#### 2.6.2. pH

The pH of the shrimp samples was determined using a digital pH meter (Mettler-Toledo GmbH, Giessen, Germany). To prepare the samples, 5 g of shrimp meat was homogenized in 25 mL of distilled water for 1 min. The homogenate was then filtered through a muslin cloth and the filtrate was collected and measured according to Ebadi et al. [3].

#### 2.6.3. Extraction of Polyphenol Oxidase

The crude polyphenol oxidase (PPO) was extracted in accordance with the method of Basiri et al. [18]. PPO from the cephalothorax was pulverized in the presence of liquid nitrogen and mixed using a pestle and mortar with 0.05 mol/L sodium phosphate buffer (pH 7.2) containing 1.0 mol/L NaCl and 0.2 g/100 mL Triton X-100 (1:3 *w*/*v* ratio). The mixture was mixed continuously for 30 min, followed by refrigerated centrifugation for 30 min at 8000× *g*. The supernatant was subjected to 40% saturation with sodium ammonium sulfate and left to stand at 4 °C for 30 min and centrifuged (12,000× *g* for 30 min at 4 °C) to collect the pellet. After centrifugation and precipitation with sodium ammonium sulfate, the resulting pellet was dialyzed using buffer overnight with buffer changes (15 volumes with three changes), and the insoluble material was collected by centrifugation (3000× *g* at 4 °C for 30 min). The resulting supernatant served as the “crude PPO extract.” To determine PPO activity, the method described by Simpson et al. [19] was followed using DOPA as the substrate. Specifically, 100 µL of the crude PPO extract was mixed in 1100 µL of the buffer solution containing 400 µL of 0.05 mol/L phosphate buffer (pH 6.0), 600 µL of 15 mmol/L DOPA, and 100 µL of deionized water. The mixture was incubated at 45 °C and the increase in absorbance at 475 nm was monitored using a UV-160 spectrophotometer (Shimadzu, Kyoto, Japan) for 3 min at 30-s intervals to measure the formation of dopachrome. PPO activity was defined as an increase in absorbance of 0.001 at 475 nm and expressed as one unit of PPO activity. The control was run in the same manner, except that deionized water was used instead of the PPO extract. The relative PPO activity was determined as the residual activity compared to the control by using the following formula:

(3) $$\mathrm{Relative}\;\mathrm{activity}\;(\%)=\frac{B}{A}\times 100$$

where A: PPO activity of control; B: PPO activity of the sample.

#### 2.6.4. Total Sulfhydryl Content

The shrimp protein isolate was prepared based on the procedure by Liu et al. [20]. The procedure involved dissolving the sample in a buffer (0.086 M Tris, 0.09 M glycine, 4 mM EDTA) at pH 8 and centrifuging at 10,000× *g* for 15 min to eliminate any insoluble protein. The total sulfhydryl content was then quantified via Ellman’s technique [21] by mixing 4.5 mL of the supernatant with 0.5 mL of Ellman’s reagent (10 mM DTNB) and the absorbance was read at 412 nm with a spectrophotometer (RF-15001, Shimadzu, Kyoto, Japan). The sulfhydryl content was quantified in µmol sulfhydryl/g protein using a molar extinction coefficient of 13,600 M^−1^ cm^−1^. In addition, the protein content of the isolate was determined using the Biuret method [22].

##### Reactive Sulfhydryl Content

The samples were placed in a solubilizing buffer (0.086 M Tris, 0.09 M glycine, 4 mM EDTA, 8 M urea) at pH 8 and then centrifuged at 10,000× *g* for 15 min to remove any insoluble protein. The reactive sulfhydryl content was determined using the DTNB assay [23]. Specifically, the supernatant (4.5 mL) was mixed with 10 mM DTNB (0.5 mL), then the absorbance was recorded at both 412 nm and 540 nm using a spectrofluorometer (RF-15001, Shimadzu, Kyoto, Japan). The reactive sulfhydryl value was calculated using the following equations:

Reactive sulfhydryl content (µmol/g) = 73.53 × (A_412_ − 1.6934 × A_532_ + 0.009932) (4)

where A_412_ and A_532_ were the absorbance 412 nm and 532 nm of the assay solution, respectively.

#### 2.6.5. Carbonyl Content

Carbonyl groups were detected by reactivity with 2,4-dinitrophenylhydrazine (DNPH). The method of Parrilla-Taylor et al. [24] was used. The samples were initially homogenized with 1 mL of 5% sulfosalicylic acid and centrifuged at 15,000× *g*, then the supernatant was discarded and the pellet was mixed with 10 mM DNPH in 2 M HCl and allowed to incubate at 25 °C for 1 h. The protein was precipitated by adding 0.5 mL of 20% TCA and centrifuged for 5 min at 15,000× *g*. The HCl was used as blanks. The resulting pellets were washed three times with a solution of ethanol and ethyl acetate (1:1, *v*/*v*) and resuspended in 6 M guanidine chlorate, followed by incubation at 37 °C for 15 min. The samples were then centrifuged for 5 min at 15,000× *g*, and the supernatant was collected for spectrophotometric measurement of protein carbonyl content at the maximum absorbance within the range of 360–401 nm. The results were reported as nmol of carbonyl proteins per gram of sample.

#### 2.6.6. Peroxide Value (PV)

The method used for lipid extraction in the shrimp was based on the approach described in the study of Bligh and Dyer [25]. A 25 g sample was homogenized with a mixture of chloroform, methanol, and distilled water (50:100:50) at 9500 rpm for 2 min at 4 °C using an IKA labortechnik homogenizer (Model T18, Bangkok, Thailand). Then, 50 mL of chloroform was added and homogenized at 9500 rpm for 1 min and 25 mL of distilled water was added and homogenized again for 30 s. The homogenate was then centrifuged at 3000 rpm at 4 °C for 15 min using a refrigerated centrifuge (Beckman Coulter, Avanti J-E Centrifuge, Fullerton, CA, USA). Finally, the chloroform phase was collected. It was then transferred into a 125 mL Erlenmeyer flask that contained about 2–5 g of anhydrous sodium sulfate, shaken well, and decanted into a round-bottom flask through a Whatman No.4 filter paper. Subsequently, the solvent was evaporated at 25 °C using an EYELA N-100 rotary evaporator (Tokyo, Japan), and the remaining solvent was removed by nitrogen flush. To determine the peroxide value (PV), the Kim et al. [6] method was adopted. The lipid sample (1.0 g) was combined with 25 mL of a solvent mixture (chloroform: acetic acid) at a ratio of 2:3 (*v*/*v*) and vigorously shaken, and 1 mL of saturated potassium iodide was introduced. After incubating the mixture in the dark for 5 min, 75 mL of distilled water was added and shaken followed by the addition of 0.5 mL of starch solution (1%, *w*/*v*) as an indicator. The mixture was then titrated against 0.01 N sodium thiosulfate solution, and the peroxide value was expressed as milliequivalents per kilogram of lipid.

#### 2.6.7. Thiobarbituric Acid Reactive Substance (TBARS)

The TBARS analysis was performed in accordance with the method of Benjakul and Bauer [26]. To begin, 1 g of minced shrimp meat was combined with 9 mL of a 15% TCA solution that contained 0.375% TBA. The resulting mixture was then subjected to heating in boiling water for 10 min, followed by cooling with running water. After this, the mixture was centrifuged for 20 min at 4000× *g*, the supernatant was collected, and its absorbance was measured at 532 nm using a UV-160 spectrophotometer (RF-15001, Shimadzu, Kyoto, Japan). The TBARS value was then determined by calculating the standard curve of MDA (ranging from 0–2 ppm) and expressed as mg MDA per kilogram of shrimp meat.

#### 2.6.8. Anisidine Value (AnV)

The Anisidine value (AnV) was determined using the method developed by Okpala et al. [27]. First, 100 mg of the lipid sample was dissolved in 25 mL of isooctane. Then, 2.5 mL of the resulting solution was mixed with 0.5 mL of 0.5% AnV in acetic acid (*w*/*v*) and kept in dark for 10 min. After that, the reaction mixture was read using a UV-Vis spectrophotometer (RF-15001, Shimadzu, Kyoto, Japan) at 350 nm and the following formula was applied to calculate the AnV value in the samples:

(5) $$\mathrm{AnV}=25\;\times \;\left(\frac{({1.2\times A}_{2})-A_{1}}{W}\right)$$

where A_1_ and A_2_ represent the absorbances measured at 350 nm before and after the addition of AnV, respectively. Furthermore, W stands for the weight of the sample (g).

#### 2.6.9. Total Oxidation Value

The totox oxidation (TOTOX) value (TV) was determined using the protocol outlined by de Abreu et al. [28]. The TV is determined by adding the peroxide value and Anisidine value (AnV), as follows:

(6) TV=2PV+AnV

### 2.7. Total Volatile Basic Nitrogen (TVB-N)

The Conway microdiffusion technique [13] was used to evaluate the TVB-N level in shrimp using 0.1 M KOH. The results were expressed in milligrams of nitrogen per 100 g of shrimp.

### 2.8. Microbiological Analyses

To conduct the microbiological tests, randomly selected shrimp samples were taken from the same tray, and each test was performed in triplicate. For these experiments, 10-g samples were transferred into sterile zipper bags containing 90 mL of peptone water and were homogenized using a stomacher at 250 rpm for 2 min. Subsequently, serial dilutions were made in test tubes containing 0.1% peptone water, and the diluted samples (0.1 mL) were then spread on the surface of dry media for microbial enumeration. Total viable plate counts (TVC) and psychrotrophic bacteria counts were determined by the pour plate method using plate count agar, which was incubated at 37 °C for 48 h and 7 °C for 10 days, respectively [29]. Enterobacteriaceae was evaluated by the spread plate method using violet red bile glucose (VRBG, Merck, Darmstadt, Germany) agar incubated at 37 °C for 24 h. Lactic acid bacteria were enumerated by the spread plate method using MRS agar incubated at 30 °C for 72 h [11]. The results were expressed in log CFU/g.

### 2.9. Statistical Analysis

The statistical analysis in this study was conducted using a completely randomized design, with all experiments performed in triplicate. Mean values were compared using analysis of variance (ANOVA) and Duncan’s multiple range test. The data were analyzed using the Statistical Package for Social Sciences (SPSS) version 6 for Windows (SPSS Inc., Chicago, IL, USA).

## 3. Results and Discussion

### 3.1. Physicochemical Properties of Different Coating Solutions

#### 3.1.1. pH, Viscosity, and Turbidity

**Figure 2:**
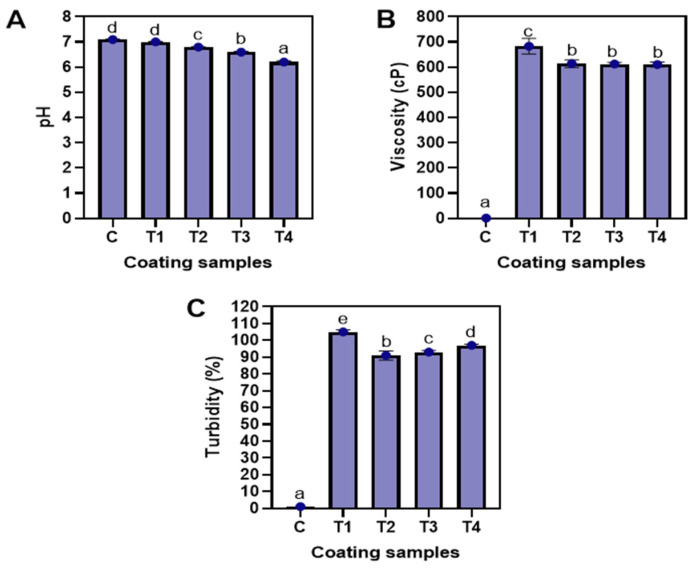
The pH (**A**), viscosity (**B**), and turbidity (**C**) of the alginate coating and alginate-based nanoparticle coating with different concentration of LPE. C: distilled water; T1: alginate coating; T2, T3, and T4: alginate-based nanoparticle coating with 0.5, 1.0, and 1.5% LPE, respectively. The different alphabets shown on the bar diagram indicate significant differences.

The pH values of the tested coating samples are illustrated in Figure 2A. On average, the pH of the coatings varied between 6.1 and 7.1. The pH of the control and T1 coating was found to be in the neutral range (*p* < 0.05), while the pH of the NP-ALG-LPE coating (T2–T4) was observed to be impacted by the LPE concentration, showing a decrease in pH as the LPE concentration increased from 0.5% to 1.5% (*p* < 0.05). Of all the samples, T4 had the lowest pH (at 6.2) value, while the control had the highest pH (at 7.1) value. Generally, the pH of longkong pericarp is slightly acidic (at 4.71) in nature [16], which may have affected the pH of the NP-ALG-LPE coatings. A study by Chen et al. [30] found that the presence of organic acids in longkong pericarp, such as glycolic acid, malic acid, and citric acid, can contribute to the low pH. Fresh longkong pericarp typically has a relatively stable pH due to its high moisture content [31]. However, the extraction process may have concentrated the naturally occurring organic acids in the pericarp, leading to a decrease in the pH of the coatings [32]. In addition, the use of ascorbic acid during extraction could have also lowered the pH of the LPE. A lower pH in the NP-ALG-LPE coatings can be beneficial as it can inhibit the growth of microorganisms. Gokoglu [33] also noted that organic acids can reduce the pH and inhibit the growth of spoilage and pathogenic bacteria. Similarly, Baek et al. [13] found that the pH of the ALG coating decreased with the addition of grapefruit seed extract due to the presence of organic compounds. The viscosity of the different coatings is shown in Figure 2B. In general, T1 had the highest viscosity, while the control had the lowest viscosity (*p* < 0.05). Among the NP-ALG-LPE coatings (T2–T4), no significant differences were observed, regardless of the LPE concentrations (*p* > 0.05). This could be due to the reduction of molecular weight of ALG caused by the ultrasonication process, which decreased the thickening properties of the ALG [34]. In general, the viscosity of the coatings is primarily determined by the material used [8]. The turbulence and cavitation effect of ultrasound causes an irreversible ordered–disordered conformation transition of ALG molecules, resulting in reduced particle size [5]. However, a lower viscosity of the coating could provide a thinner film over the food samples. Pilon et al. [14] stated that a thin layer over the product has better barrier properties during prolonged storage. As noted by Rodriguez-Turienzo et al. [35], the viscosity of a whey protein isolate-based coating was found to be decreased when ultrasonication treatment was applied in the coating emulsion and thus attributed to the formation of nano-sized particles. The turbidity levels of the different coatings are shown in Figure 2C. The NP-ALG-LPE coatings (T2–T4) that were subjected to ultrasonication had lower turbidity compared to T1 (*p* < 0.05). This can be attributed to the reduction of starch granules in the ALG caused by the ultrasonication process, which leads to a decrease in opacity [36]. The overall transparency of NP-ALG-LPE coatings was improved as a result of ultrasonication. The solubility of ALG increased in samples T2, T3, and T4 due to ultrasonication, while T1 without ultrasonication had more undissolved particles [37]. Jambrak et al. [38] postulated that ultrasonication destroyed the crystalline region of starch granules, resulting in higher solubility of corn starch. Among the NP-ALG-LPE coatings (T2–T4), turbidity increased with augmenting concentrations of LPE from 0.5% to 1.5%, respectively (*p* < 0.05). Generally, the higher turbidity of the coating negatively impacts the product’s appearance, thus reducing marketability [5]. Increasing the coating ingredients promoted more polymers to be present in the coating, giving rise to greater light scattering [39]. Moreover, the higher concentration of LPE (1.5%) in T4 had a larger particle size. Therefore, more light scattering increased the turbidity of the coating.

#### 3.1.2. Whiteness Index, Particle Size, and Polydispersity Index

**Figure 3:**
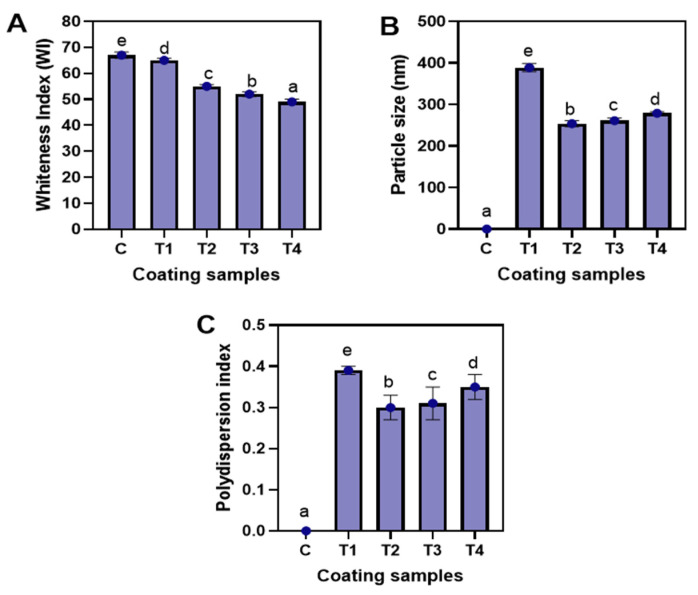
Whiteness index (**A**), particle size (**B**), polydispersity index (**C**) of the alginate coating and alginate-based nanoparticle coating with different concentration of LPE. C: distilled water; T1: alginate coating; T2, T3, and T4: alginate-based nanoparticle coating with 0.5, 1.0, and 1.5% LPE, respectively. The different alphabets shown on the bar diagram indicates significant differences.

Figure 3A displays the whiteness index (WI) of the tested coating samples. Generally, coatings with higher WI have a more desirable commercial appeal. The T1 coating had a higher WI than the NP-ALG-LPE coatings (T2–T4) with a significant difference (*p* < 0.05). The decrease in WI of NP-ALG-LPE coatings (T2–T4) was attributed to the yellowish-brownish color of LPE [4]. As the concentration of LPE increased from 0.5–1.5%, the WI significantly decreased (*p* < 0.05). According to Lichanporn et al. [31], brown pigments are mainly present in the pericarp area of longkong fruits. Therefore, the higher amount of LPE (1.5%) used in the coating resulted in a reduction of whiteness. Figure 3B illustrates the particle size of various coatings. In general, the NP-ALG-LPE coatings (T2–T4) had smaller particle sizes than T1 with a significant difference (*p* < 0.05). The shear force created by ultrasonic cavitation caused a reduction in the particle size of polymers in the coatings [40]. Among the NP-ALG-LPE coatings, the particle size increased as the concentration of LPE increased from 0.5% to 1.5% with a significant difference (*p* < 0.05). T2 had the smallest particle size, followed by T3 and T4 (*p* < 0.05). The particle size of NP-ALG-LPE coatings ranged from 218 to 260 nm. This is in line with the findings of Baek et al. [13] and Lin et al. [41], who reported particle sizes of 206 nm and 247 nm for ultrasonicated ALG and chitosan-based coatings, respectively. Figure 3C shows the polydispersity index (PDI) of the tested coating samples. The PDI measures the heterogeneity of a coating based on its particle size [42]. Typically, a lower PDI indicates a more uniform distribution and smaller size of polymers in the coating [43,44]. In general, NP-ALG-LPE coatings (T2–T4) had a lower PDI compared to T1 with a significant difference (*p* < 0.05). This difference was likely due to the disruption of structural integrity, induction of dissociation, and degradation of ALG molecules caused by the cavitation effect of ultrasonication [5], resulting in a reduction in the size of ALG molecules. This implies that ultrasonicated NP-ALG-LPE coatings have a homogenous dispersion of particles in the medium. Among the NP-ALG-LPE coatings, T2 had the lowest PDI. The increase in LPE concentration from 0.5% to 1.5% resulted in larger particle size and uneven distribution, leading to a higher PDI in T4 coating, followed by T3, as compared to T2 (*p* < 0.05). This is consistent with the particle size results shown in Figure 3B. Thus, the application of ultrasound decreases the particle size and PDI of the coating.

### 3.2. Shelf-Life Analysis of Refrigerated Shrimp Using Different ALG-Based Coatings

#### 3.2.1. Physicochemical Properties

**Figure 4:**
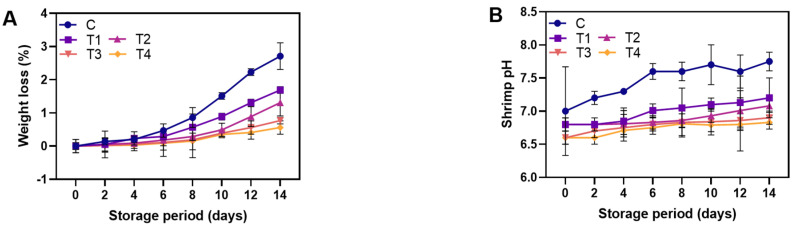
Changes in weight loss (**A**) and pH (**B**) of the shrimps coated with different coatings during storage of 14 days at 4 °C. C: shrimp dipped in distilled water as control; T1: shrimp coated with alginate coating; T2, T3, and T4: shrimp coated with 0.5, 1.0, and 1.5% LPE, respectively.

Figure 4A shows the weight loss of control and coated shrimps during storage at 4 °C. Generally, weight loss represents moisture loss of fresh food, impacting both economic benefits and product quality, making it a crucial factor for marketability [8,12]. Weight loss in all samples rose as storage days progressed (*p* < 0.05). The probable cause of weight loss in the shrimp sample was due to the denaturation and degradation of protein through autolysis and microbial enzymes, resulting in structural breakdown during storage [32]. Consequently, the water-holding capacity of shrimp muscle significantly decreased with storage time. At the end of storage, the T4-coated sample had the lowest weight loss as compared to the other samples. No significant difference was noted between the control and T1-coated sample until day 6 of storage (*p* > 0.05). Afterward, the T1-coated sample displayed a noticeable decrease in moisture loss compared to the control (*p* < 0.05) due to the barrier properties of ALG, which reduced moisture loss during storage [13]. The semi-permeable coating layer acted as a barrier to the flow of O_2_, CO_2_, and H_2_O, thus reducing moisture loss [6]. Moreover, the protein–polysaccharide complex formed between ALG, and the muscle protein of shrimp could enhance the overall water-holding capacity of shrimp during storage [45]. In addition to this, dehydration needs to be considered because it is one of the important quality parameters during refrigerated storage. According to Song et al. [8], the primary reason for the decrease in dehydration of products coated with ALG is that the gel coating serves as a sacrificial agent. Thus, moisture in the gel evaporates before any substantial desiccation occurs in coated food. Additionally, no significant difference was observed among NP-ALG-LPE coated shrimp samples until the 10th day of storage. However, at the end of storage, the T4-coated sample had the lowest weight loss, followed by T3, when compared to T2 (*p* < 0.05). This was due to the formation of an additional layer on the shrimps, providing further resistance to mass transfer and slowing the increase in water loss after a certain storage period [39]. As a result, the addition of LPE to the ALG coating on shrimp effectively reduced moisture loss during prolonged storage. pH is recognized to be one of the important indicators for identifying the changes in pH of control and coated shrimps during storage microbial spoilage in seafood or aquatic products [32]. On day 0, the samples’ pH was 6.6 to 6.9, consistent with previous reports [1,6]. The slightly lowered pH of the NP-ALG-LPE-coated samples (T2–T4) was due to the reduced pH of the coatings with the addition of LPE (0.5–1.5%). This was in agreement with the pH of the NP-ALG-LPE coatings (Figure 4B). Overall, pH values were gradually increased in all samples over the storage period. At the end of storage, the lowest pH was observed in the T4-coated shrimp, while the control sample had the highest pH (*p* < 0.05). The rise in pH was associated with the accumulation of alkaline compounds, primarily produced due to microbial activity. Ebadi et al. [3] noted that endogenous enzymes and microorganisms breaking down protein in shrimp produce volatile bases like ammonia and trimethylamine, causing an increase in shrimp muscle pH during storage [46]. T4-coated shrimp had a smaller increase in pH, which matched the controlled growth of the microorganisms. In general, shrimp is considered unacceptable if the pH exceeds 7.6 [47]. The control sample reached this limit on day 6, while coated shrimp remained within acceptable limits throughout storage. Among NP-ALG-LPE-coated shrimp samples (T2–T4), the pH value of the samples was strongly decreased with increasing LPE concentration (*p* < 0.05). This suggests that LPE may help to slow microbial growth, thus reducing spoilage and decomposition. Shrimp coated with T4, which contains 1.5% LPE, showed lower pH due to the inhibitory effect of LPE against microbial action. This is because LPE contains high levels of polyphenols [31], and their activity against microbes is dependent on concentration. Polyphenols interact with microbial membranes, altering their permeability and functions and leading to cell death [48,49].

#### 3.2.2. Brown Pigment Enzyme Activities

**Figure 5:**
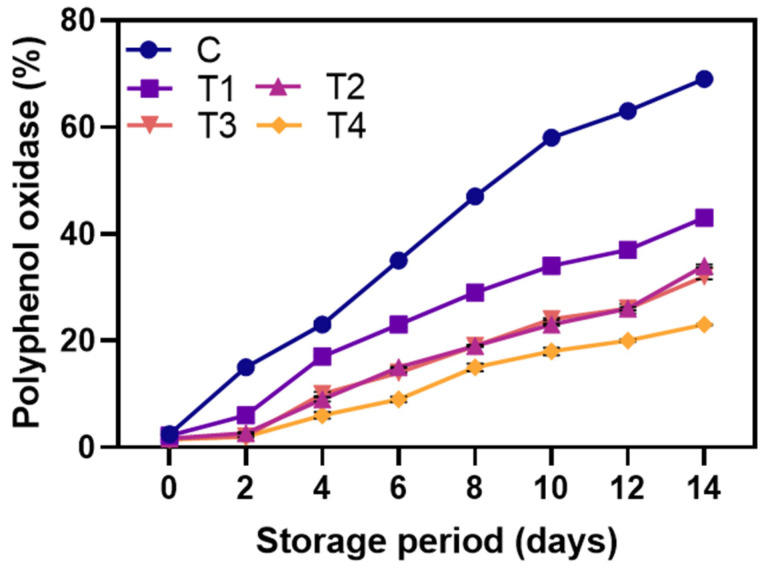
Changes in polyphenol oxidase of the shrimps coated with different coatings during storage of 14 days at 4 °C. C: shrimp dipped in distilled water as control; T1: shrimp coated with alginate coating; T2, T3, and T4: shrimp coated with 0.5, 1.0, and 1.5% LPE, respectively.

Figure 5A displays the levels of PPO activities in both control and coated shrimp samples during storage at 4 °C. PPO is a major contributor to browning in the shrimp samples during prolonged storage. Enzymatic browning, also known as melanosis, affects the quality and acceptance of shrimps. PPO is the main cause of browning, converting colorless quinones into dark pigments [50]. PPO triggers enzymatic browning by catalyzing the reaction between substrates, oxygen, phenolic compounds, and reaction by-products, causing black spot formation [6]. Among the samples, the control exhibited the highest PPO activity. This was attributed to the basic mechanism of melanosis, in which PPO converts colorless monophenols into diphenols, which then react with oxygen to form highly colored quinones, leading to the formation of brown polymers through reaction with amino acids [51]. Shrimp coated with T4 had the lowest PPO activity during storage due to the higher concentration of LPE (1.5%) compared to T3 (1.0%) and T2 (0.5%) (*p* < 0.05). The inhibition of PPO activity by phenolic compounds can occur through various mechanisms, including direct inhibition of PPO, scavenging of oxygen, and reduction of quinones back to diphenols to prevent melanin formation [46]. Balti et al. [52] found that shrimps coated with microalgal exopolysaccharides enriched with 1.5% red seaweed extract (contains rich sources of polyphenols) reduced PPO activity more effectively than those coated with 0.5% and 1.0% extract during cold storage. Hence, the results suggest that coating shrimp with 1.5% LPE effectively inhibits PPO activity during storage, making it a promising natural inhibitor.

#### 3.2.3. Protein Oxidation

**Figure 6:**
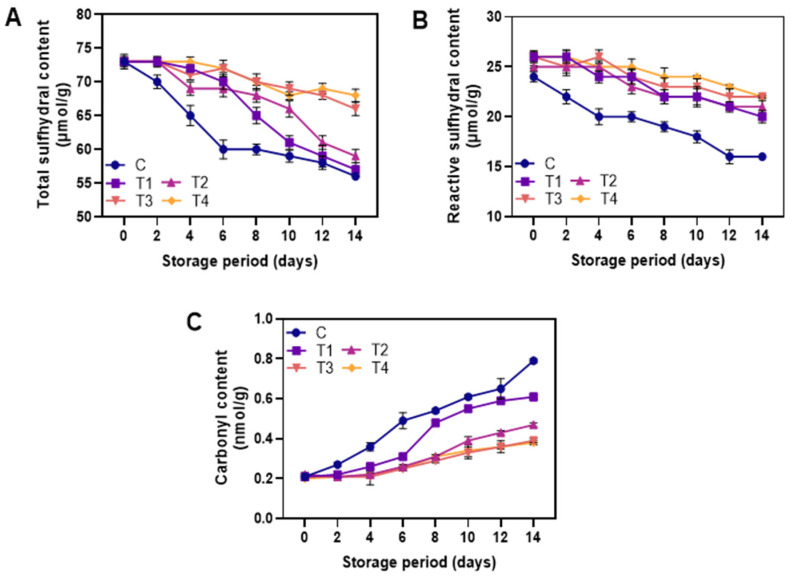
Changes in total sulfhydral content (**A**), reactive sulfhydral content (**B**), and carbonyl content (**C**) of the shrimps coated with different coatings during storage of 14 days at 4 °C. C: shrimp dipped in distilled water as control; T1: shrimp coated with alginate coating; T2, T3, and T4: shrimp coated with 0.5, 1.0, and 1.5% LPE, respectively.

Figure 6A,B depicts the total and reactive sulfhydryl groups of the control and coated shrimps during storage at 4 °C. Sulfhydryl is a highly active group found in myofibrillar protein, which possesses weak secondary bonds [53]. Overall, sulfhydryl content decreased in all samples with increasing storage time (*p* < 0.05). This indicated that prolonged storage of shrimp had a significant effect on protein oxidation. However, the decrease of sulfhydryl groups was found lower in coated samples (T1–T4) when compared to the control (*p* < 0.05). Morachis-Valdez et al. also reported that carp fillets coated with chitosan showed less reduction in sulfhydryl content than uncoated ones during 5 months of storage at −18 °C [54]. In general, protein oxidation leads to a decrease in sulfhydryl groups, which become disulfides [55]. Additionally, muscle protein denaturation and aggregation are related to disulfide bonds [4]. Among the NP-ALG-LPE coatings (T2–T4), T2 showed the greatest reduction while T4 showed the least loss in the sulfhydryl groups. The loss of sulfhydryl groups affects the structural, functional, and nutritional properties of shrimp muscle protein [56]. This negative effect can be reduced with the use of LPE which showed a superior protective effect and showed greater stability during storage. The carbonyl content in control and coated shrimps during storage at 4 °C is shown in Figure 6C. Carbonyl is a marker of protein oxidation, measured using DNPH (2,4-Dinitrophenylhydrazine) [57]. Protein oxidation decreases shrimp quality and nutrition due to the loss of essential amino acids and reduced digestibility [58]. Carbonyl content increased in all samples during storage (*p* < 0.05), indicating oxidative damage to amino acid side chains, such as lysine, proline, arginine, and histidine [59]. On day 4, the control sample had higher carbonyl content than all coated samples (T1–T4), which increased over time. Shrimp coated with NP-ALG-LPE (T2–T4) significantly prevented the level of protein oxidation as compared with the ALG coating alone (T1) and control (*p* < 0.05). However, the addition of LPE in the NP-ALG coating (T2–T4) did not significantly affect the carbonyl content in the samples until day 8. At the end of storage, samples coated with T3 and T4 had lower carbonyl content than T2 (*p* < 0.05). Secondary products of lipid oxidation, such as aldehydes (e.g., malondialdehyde and 4-hydroxy-2-nonenal) or ketones, can react with amino acid residues through covalent bonds, known as Michael addition reactions, leading to indirect oxidation of protein [60]. Hence, LPE could scavenge this oxidation process in the stored shrimps as it contains an abundant level of polyphenols which act as a primary scavenger for oxidation and oxidation-induced byproducts.

#### 3.2.4. Lipid Oxidation

**Figure 7:**
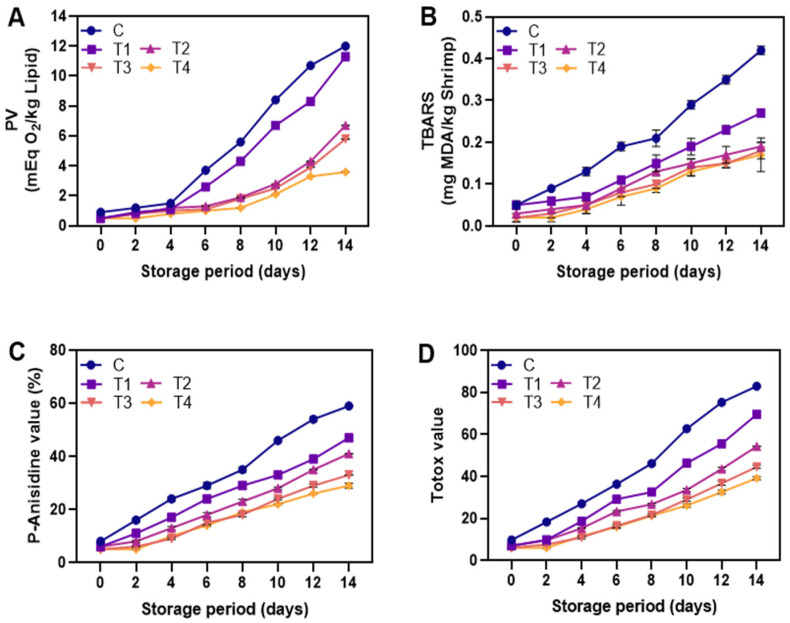
Changes in PV (**A**), TBARS (**B**), p-anisidine value (**C**), and totox value (**D**) of the shrimp coated with different coating during storage of 14 days at 4 °C. C: shrimp dipped in distilled water as control; T1: shrimp coated with alginate coating; T2, T3, and T4: shrimp coated with 0.5, 1.0, and 1.5% LPE, respectively.

The peroxide value (PV) is a measure of major lipid oxidation products (hydroperoxides) in a sample [61]. The abstraction of hydrogen from fatty acid double bonds produces fatty acid-free radicals, which further react with oxygen to form hydroperoxides [50]. All samples showed a gradual increase in PV (Figure 7A) level over storage (*p* < 0.05), indicating oxidation of fatty acids in shrimp muscle, producing hydroperoxides or peroxides [52]. On day 6 of storage, a significant difference was observed between control and coated samples (*p* < 0.05), with control samples having higher PV, indicating greater lipid oxidation. Coated samples, however, showed lower PV during storage (*p* < 0.05), attributed to the oxygen barrier capacity of ALG reducing oxygen diffusion and preventing lipid oxidation [6]. Shrimp coated with T4 had the lowest PV compared to control and T1, T2, and T3 throughout storage (*p* < 0.05). In addition, PV did not exceed the acceptable limit of 18–20 meq/kg [62]. This showed that the antioxidant activity of LPE in preventing lipid oxidation was concentration dependent. Lipid oxidation is generally accelerated during storage due to high levels of polyunsaturated fatty acids in crustacean cell membranes [11], but phenolic compounds in LPE might scavenge free radicals, reducing lipid oxidation by lowering lipid radicals [35]. Thus, shrimp coated with T4, with the lowest PV, showed the best protection against lipid oxidation. The increase in TBARS (a measure of lipid oxidation) was due to partial oxidation and dehydration of unsaturated fatty acids [32]. All samples showed a rise in TBARS over 14 days of storage (*p* < 0.05) (Figure 7B). Shrimps are susceptible to lipid oxidation, which can occur through autoxidation, photosensitized oxidation, or enzymatic reactions, such as lipoxygenase, peroxidase, and microbial enzymes [17]. In the present study, shrimp coated with 1.5% LPE (T4) showed the lowest TBARS level compared to T1 and control samples at all storage times (*p* < 0.05), followed by T3 and T2. The decrease in TBARS values was consistent with the decrease in peroxide values (PV), indicating the ability of LPE to scavenge free radicals and prevent the formation of secondary oxidation products. The NP-ALG coating acted as a barrier to oxygen permeation and a carrier for the antioxidants in LPE, thus reducing the production of secondary oxidation products in shrimp [13]. Notably, T4 had the highest amount of LPE (1.5%) in its coating, thus resulting in the lowest TBARS value. Polyphenols, which are abundant in longkong fruit, have a strong reducing capacity and are known to retard and inhibit lipid oxidation [63]. Souza et al. [64] demonstrated that a polyphenol-rich leaf extract from an Amazonian plant act as a powerful antioxidant in human LDL protein by reducing TBARS levels. Furthermore, the TBARS value of all groups was below the acceptable limit (1–2 mg MDA/kg) [11], consistent with the study by Dehghani et al. [65]. Thus, shrimp coated with T4 showed higher stability against lipid oxidation. The secondary oxidation products of shrimp were measured using AnV, which detects non-volatile oxidation products, such as aldehydes and ketones [27]. AnV reacts with oxidation products to produce a yellow product [66]. The trend of AnV was similar to other lipid oxidation product assays in this study. The control had higher AnV compared to coated samples, with T4 having the lowest AnV (Figure 7C). This suggests that LPE in ALG coating reduces secondary oxidation product formation, especially in non-volatile compounds. The results from AnV are consistent with PV and TBARS, confirming that LPE in ALG coating is an effective way to maintain shrimp quality. The totox value measures the total lipid degradation products and indicates the oxidative stage of a product [67,68]. The totox value combines primary and secondary oxidation products and is commonly used in the food industry. The totox value increased over time for all samples, with the highest value found in the control (Figure 7D). A lower totox value indicates better shrimp quality. Among the NP-ALG-LPE-coated shrimps, T4 showed the lowest totox value compared to T2 and T3. Generally, the coating application on the food surface can cause the migration of compounds from the coating into the food [69]. As a result, coating with a higher LPE content (1.5%) resulted in higher levels of migrated substances and a stronger antioxidant effect in the shrimp, providing greater stability against lipid oxidation.

#### 3.2.5. TVB-N

**Figure 8:**
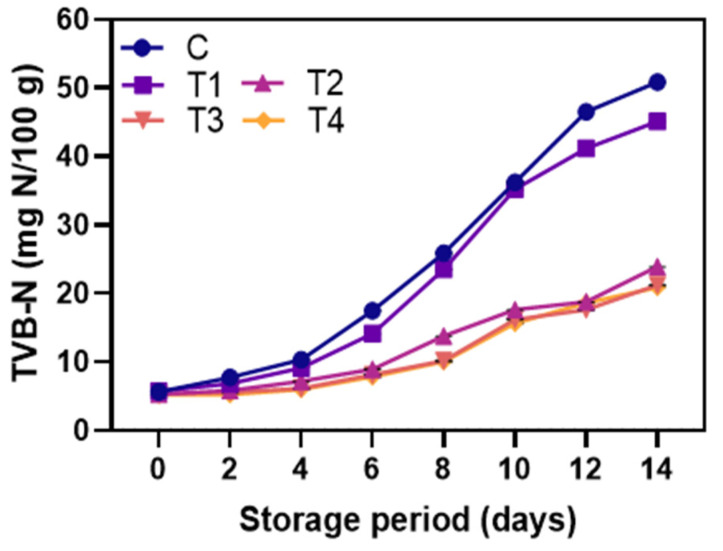
Changes in TVB-N value of the shrimps coated with different coatings during storage of 14 days at 4 °C. C: shrimp dipped in distilled water as control; T1: shrimp coated with alginate coating; T2, T3, and T4: shrimp coated with 0.5, 1.0, and 1.5% LPE, respectively.

The results of TVB-N levels in control and coated shrimp during storage at 4 °C are shown in Figure 8. TVB-N indicates decomposition of protein by microorganisms or endogenous enzymes in foods, particularly seafoods [70]. An analytical technique called TVB-N (Total Volatile Basic Nitrogen) measures the levels of nitrogenous chemicals in shrimp or seafood products, which reveals the level of freshness. TVB-N provides the total base volatile nitrogen content that begins to accumulate in the tissues with degradation during shrimp storage [65]. TVB-N, the combination of ammonia (from amino acid degradation), di-methylamine (generated by self-degrading enzymes), and trimethylamine (result of spoilage bacteria), generally rises with bacterial growth, enzyme degradation, or a combination of both during storage [71]. At day 0, TVB-N levels of all samples were below 10 mg N/100 g, showing freshness of the raw material. The control’s TVB-N content rapidly rose with storage time (*p* < 0.05), reaching 58.4 mg N/100 g on day 14. Shrimp coated with the NP-ALG-LPE coatings (T2–T4) showed a slower increase in TVB content compared to the control and T1-coated samples during storage (*p* < 0.05). Additionally, samples coated with T4 had lower TVB content compared to those coated with T2 and T3 (*p* < 0.05). The European Commission considers TVB-N levels of 30–35 mg N/100 g as the upper acceptable limit [72]. The amount of TVB-N increased as a sum of ammonia, dimethylamine, trimethylamine, and volatile amine compounds. A small amount of ammonia is generally found during the first weeks of storage, and the total volatile alkalinity is slowly increased during storage. This may be caused by the amine removal process (deamination) of amino acids [73]. This study found that the NP-ALG-LPE-coated samples (T2–T4) had remained within acceptable limits at the end of storage. The reduced TVB-N content in the NP-ALG-LPE-coated samples was due to either reduced degradation of non-protein nitrogen compounds or slowed bacterial growth, or both. This shows LPE’s antibacterial properties. Olatunde et al. [74] also found that coconut husk extract reduced the increase in TVB in Asian sea-bass slices during 12 days of storage at 4 °C. Moreover, the quality and shelf-life of shrimp coated in active edible coatings made of gelatin and orange peel essential oil were determined. The shelf-life of shrimp was evaluated over a 14-day storage period by TVB-N analysis. Compared to the control group, the addition of orange peel essential oil in the edible gelatin coating improved the quality and prolonged the shelf-life of the shrimp. The incorporation of orange peel essential oil helped in preserving the chemical and microbial quality of the shrimp [75].

#### 3.2.6. Microbial Analysis

**Figure 9:**
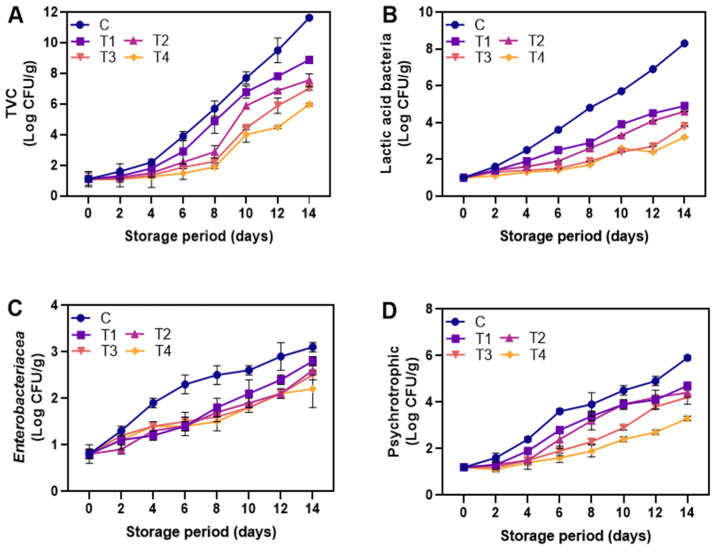
Changes in TVC (**A**), Lactic acid bacteria (**B**), Enterobacteriacea (**C**), and Psychrotrophic bacteria (**D**) of the shrimps coated with different coating during storage of 14 days at 4 °C. C: shrimp dipped in distilled water as control; T1: shrimp coated with alginate coating; T2, T3, and T4: shrimp coated with 0.5, 1.0, and 1.5% LPE, respectively.

The initial total viable count (TVC) of the shrimp was around 2.4–2.9 log CFU/g, similar to the result of 2.5–3 log CFU/g reported by Mohebi et al. [76]. During 14 days of storage, a general increase in TVC was observed in all samples (*p* < 0.05), with the highest increase seen in the control (Figure 9A), reaching 23.15 log CFU/g on the 14th day (*p* < 0.05). Dipping the shrimp in ALG reduced the increasing trend of TVC but adding LPE significantly enhanced the microbial inhibitory effect (*p* < 0.05). There were no significant differences among NP-ALG-LPE-coated samples (T2–T4) until day 4 of storage (*p* > 0.05), but the T4-coated sample had the lowest TVC among all tested samples at the end of storage (*p* < 0.05). This indicates that the inhibitory effect increased with increasing LPE concentration (0.5–1.5%) (*p* < 0.05). Phytochemical compounds can damage bacterial cells by disrupting cell membranes and precipitating cell protein, causing death [52]. Kim et al. [6] found that shrimp coated with chitosan-alginate containing grape seed extract reduced TVC during 15 days of storage at 4 °C. Liu et al. [77] reported that an alginate-calcium coating with methanol extract from citrus fruit effectively reduced the increasing trend of TVC compared to those coated with 1% chitosan. Lactic acid bacteria (LAB) are facultative anaerobes and form a significant part of the natural microbiota in seafood stored in anaerobic conditions [78]. On the initial day, the count of lactic acid bacteria (LAB) in all samples was 1.0 log CFU/g (Figure 9B). During storage, there was an observed increase in LAB count in all samples (*p* < 0.05). However, samples coated with T1–T4 exhibited a reduced increment compared to the control (*p* < 0.05). The NP-ALG coating had an inhibitory effect on LAB growth, which was intensified by the addition of LPE at a different concentration. T1, T2, T3, and T4 reduced the LAB count by 2, 2.16, 2.67, and 3.15 log CFU/g compared to the control, respectively. Khaledian et al. [11] found that shrimp coated with a tragacanth gum-based coating containing lime peel extract inhibited LAB growth during 10-day storage at 4 °C. Among the NP-ALG-LPE-coated shrimp samples, T4 had a lower LAB count due to higher LPE concentration (1.5%) and greater antimicrobial effect. This can be attributed to the presence of terpenoids and lansiosides, which are the two major antibacterial substances found in longkong fruit [79,80]. A smoked eel fillet coated with carboxy methylcellulose containing rosemary extract (200–800 ppm) also showed a decrease in LAB count [81]. Enterobacteriaceae are used as a measure of hygiene [11]. Their presence in seafood, especially in cases of contaminated water or delayed chilling after capture, increases the likelihood of spoilage [82]. The initial count of Enterobacteriaceae in all samples was 0.95 log CFU/g (Figure 9C). During storage, the count of this bacteria increased continuously in all samples (*p* < 0.05). Among the NP-ALG-LPE-coated shrimp samples (T2–T4), T4 had the lowest count of Enterobacteriaceae as compared to the control (*p* < 0.05). T4 was effective in slowing the growth of Enterobacteriaceae, followed by T3 and T2 (*p* > 0.05). This suggests that the antimicrobial effect of LPE was dose dependent. Alsaggaf et al. [83] found that shrimp coated with chitosan enriched with pomegranate peel extract reduced the microbial count during 30-day storage at 4 °C, with higher PPE concentration (0.5–2.0%) improving the antimicrobial activity. At the beginning of shrimp storage, the psychrotrophic bacteria count (PBC) of all samples was recorded at 1.85 log CFU/g (Figure 9D). Over time, the PBC of the control sample continued to rise (*p* < 0.05), but the count decreased in shrimp coated with ALG (T1) (*p* < 0.05). Gram-negative psychrotrophic bacteria are a key cause of spoilage in iced and/or refrigerated seafood [84]. Shrimp coated with NP-ALG-LPE coatings had an even lower level of PBC as compared to T1 and the control (*p* < 0.05), which confirms the antibacterial activity of LPE. Other studies have also found similar results in shrimp samples treated with green tea extract [46] and fish fillets treated with microalgal exopolysaccharides and red seaweed extract [52]. The use of NP-ALG enriched with LPE as an active edible coating is a promising solution for maintaining the quality of shrimp during refrigerated storage by limiting the growth of multiple bacteria.

## 4. Conclusions

The study investigated the impact of adding LPE (0.5–1.5%) to the ALG coating, using the ultrasonication process to convert the ALG-LPE coating to become nano-sized, and tested various physiochemical properties in the coating emulsion and as well as on the shrimp samples to control the quality loss and prolong the shelf-life for up to 14 days at 4 °C. The results showed that LPE addition to a coating emulsion significantly increased the viscosity, turbidity, particle size, and polydispersity index of the coating material while lowering the pH and whiteness index. The best results were observed when 1.5% LPE was added to the NP-ALG-based coating and tested shrimp samples, which resulted in lower pH, weight loss, and polyphenol oxidase activity, as well as stronger antioxidant effects against protein and lipid oxidation in the shrimp samples. The study also found that the microbial count, including total viable count, lactic acid bacteria, Enterobacteriaceae, and psychrotrophic bacterial count, was lower in the shrimps coated with the ALG-LPE coating. Thus, the results suggest that the addition of 1.5% LPE to the ALG coating can effectively maintain the quality of shrimp for up to 14 days of storage and is a good alternative to synthetic preservatives. In summary, synergistic effect of an alginate-based nanoparticle coating and LPE can serve as promising approach for the quality maintenance of seafood during storage. This finding could be potentially beneficial for food industries to extend the shelf-life of food products without the addition of synthetic preservatives. Further research is anticipated to rationally designed edible coatings with precisely adjusted coating properties for the effective enhancement of food products’ quality and storability.

## Data Availability

Data is contained within the article.

## References

[1] Sabu S., Xavier K.M., Sasidharan A. (2021). Efficacy of pomegranate phenolic extract and chitosan as an edible coating for shelf life extension of Indian white shrimp during refrigerated storage. J. Packag. Technol. Res..

[2] Licciardello F., Kharchoufi S., Muratore G., Restuccia C. (2018). Effect of edible coating combined with pomegranate peel extract on the quality maintenance of white shrimps (*Parapenaeus longirostris*) during refrigerated storage. Food Packag. Shelf Life.

[3] Ebadi Z., Khodanazary A., Hosseini S.M., Zanguee N. (2019). The shelf life extension of refrigerated Nemipterus japonicus fillets by chitosan coating incorporated with propolis extract. Int. J. Biol. Macromol..

[4] Nagarajan M., Rajasekaran B., Benjakul S., Venkatachalam K. (2021). Influence of chitosan-gelatin edible coating incorporated with longkong pericarp extract on refrigerated black tiger Shrimp (*Penaeus monodon*). Curr. Res. Food Sci..

[5] Sharifimehr S., Soltanizadeh N., Hossein Goli S.A. (2019). Effects of edible coating containing nano-emulsion of Aloe vera and eugenol on the physicochemical properties of shrimp during cold storage. J. Sci. Food Agric..

[6] Kim J.-H., Hong W.-S., Oh S.-W. (2018). Effect of layer-by-layer antimicrobial edible coating of alginate and chitosan with grapefruit seed extract for shelf-life extension of shrimp (*Litopenaeus vannamei*) stored at 4 C. Int. J. Biol. Macromol..

[7] Alexandre S., Vital A.C.P., Mottin C., do Prado R.M., Ornaghi M.G., Ramos T.R., Guerrero A., Pilau E.J., do Prado I.N. (2021). Use of alginate edible coating and basil (*Ocimum* spp.) extracts on beef characteristics during storage. J. Food Sci. Technol..

[8] Song Y., Liu L., Shen H., You J., Luo Y. (2011). Effect of sodium alginate-based edible coating containing different anti-oxidants on quality and shelf life of refrigerated bream (*Megalobrama amblycephala*). Food Control.

[9] Maqsood S., Benjakul S., Abushelaibi A., Alam A. (2014). Phenolic compounds and plant phenolic extracts as natural antioxidants in prevention of lipid oxidation in seafood: A detailed review. Compr. Rev. Food Sci. Food Saf..

[10] Venkatachalam K. (2018). Influence of prolonged salting on the physicochemical properties of duck egg white. Braz. Arch. Biol. Technol..

[11] Khaledian S., Basiri S., Shekarforoush S.S. (2021). Shelf-life extension of pacific white shrimp using tragacanth gum-based coatings containing Persian lime peel (*Citrus latifolia*) extract. LWT.

[12] Xing Y., Li W., Wang Q., Li X., Xu Q., Guo X., Bi X., Liu X., Shui Y., Lin H. (2019). Antimicrobial nanoparticles incorporated in edible coatings and films for the preservation of fruits and vegetables. Molecules.

[13] Baek J.H., Lee S.-Y., Oh S.-W. (2021). Enhancing safety and quality of shrimp by nanoparticles of sodium alginate-based edible coating containing grapefruit seed extract. Int. J. Biol. Macromol..

[14] Pilon L., Spricigo P.C., Miranda M., de Moura M.R., Assis O.B.G., Mattoso L.H.C., Ferreira M.D. (2015). Chitosan nanoparticle coatings reduce microbial growth on fresh-cut apples while not affecting quality attributes. Int. J. Food Sci. Technol..

[15] Josewin S.W., Ghate V., Kim M.-J., Yuk H.-G. (2018). Antibacterial effect of 460 nm light-emitting diode in combination with riboflavin against Listeria monocytogenes on smoked salmon. Food Control.

[16] Venkatachalam K. (2015). The different concentrations of citric acid on inhibition of Longkong pericarp browning during low temperature storage. Int. J. Fruit Sci..

[17] Khazaei N., Esmaiili M., Emam-Djomeh Z. (2016). Effect of active edible coatings made by basil seed gum and thymol on oil uptake and oxidation in shrimp during deep-fat frying. Carbohydr. Polym..

[18] Basiri S., Shekarforoush S.S., Aminlari M., Akbari S. (2015). The effect of pomegranate peel extract (PPE) on the polyphenol oxidase (PPO) and quality of Pacific white shrimp (*Litopenaeus vannamei*) during refrigerated storage. LWT-Food Sci. Technol..

[19] Simpson B.K., Marshall M.R., Otwell W.S. (1987). Phenol oxidase from shrimp (*Penaeus setiferus*): Purification and some properties. J. Agric. Food Chem..

[20] Liu G., Xiong Y.L. (1996). Contribution of lipid and protein oxidation to rheological differences between chicken white and red muscle myofibrillar proteins. J. Agric. Food Chem..

[21] Ellman G.L. (1959). Tissue sulfhydryl groups. Arch. Biochem. Biophys..

[22] Robinson H.W., Hogden C.G. (1940). The biuret reaction in the determination of serum proteins. 1. A study of the conditions necessary for the production of a stable color which bears a quantitative relationship to the protein concentration. J. Biol. Chem..

[23] Xia X., Kong B., Liu Q., Liu J. (2009). Physicochemical change and protein oxidation in porcine longissimus dorsi as influenced by different freeze–thaw cycles. Meat Sci..

[24] Parrilla-Taylor D.P., Zenteno-Savín T., Magallón-Barajas F.J. (2013). Antioxidant enzyme activity in pacific whiteleg shrimp (*Litopenaeus vannamei*) in response to infection with white spot syndrome virus. Aquaculture.

[25] Bligh E.G., Dyer W.J. (1959). A rapid method of total lipid extraction and purification. Can. J. Biochem. Physiol..

[26] Benjakul S., Bauer F. (2001). Biochemical and physicochemical changes in catfish (*Silurus glanis Linne*) muscle as influenced by different freeze–thaw cycles. Food Chem..

[27] Okpala C.O.R., Choo W.S., Dykes G.A. (2014). Quality and shelf life assessment of Pacific white shrimp (Litopenaeus vannamei) freshly harvested and stored on ice. LWT-Food Sci. Technol..

[28] de Abreu D.P., Losada P.P., Maroto J., Cruz J. (2011). Lipid damage during frozen storage of Atlantic halibut (Hippoglossus hippoglossus) in active packaging film containing antioxidants. Food Chem..

[29] Rezaeifar M., Mehdizadeh T., Mojaddar Langroodi A., Rezaei F. (2020). Effect of chitosan edible coating enriched with lemon verbena extract and essential oil on the shelf life of vacuum rainbow trout (*Oncorhynchus mykiss*). J. Food Saf..

[30] Chen X., Ren L., Li M., Qian J., Fan J., Du B. (2017). Effects of clove essential oil and eugenol on quality and browning control of fresh-cut lettuce. Food Chem..

[31] Lichanporn I., Srilaong V., Wongs-Aree C., Kanlayanarat S. (2009). Postharvest physiology and browning of longkong (Aglaia dookkoo Griff.) fruit under ambient conditions. Postharvest Biol. Technol..

[32] Farajzadeh F., Motamedzadegan A., Shahidi S.-A., Hamzeh S. (2016). The effect of chitosan-gelatin coating on the quality of shrimp (Litopenaeus vannamei) under refrigerated condition. Food Control.

[33] Gokoglu N. (2019). Novel natural food preservatives and applications in seafood preservation: A review. J. Sci. Food Agric..

[34] Bonilla J., Atarés L., Vargas M., Chiralt A. (2012). Effect of essential oils and homogenization conditions on properties of chitosan-based films. Food Hydrocoll..

[35] Rodriguez-Turienzo L., Cobos A., Diaz O. (2012). Effects of edible coatings based on ultrasound-treated whey proteins in quality attributes of frozen Atlantic salmon (*Salmo salar*). Innov. Food Sci. Emerg. Technol..

[36] Cheng J., Cui L. (2021). Effects of high-intensity ultrasound on the structural, optical, mechanical and physicochemical properties of pea protein isolate-based edible film. Ultrason. Sonochemistry.

[37] Amiri A., Sharifian P., Soltanizadeh N. (2018). Application of ultrasound treatment for improving the physicochemical, functional and rheological properties of myofibrillar proteins. Int. J. Biol. Macromol..

[38] Jambrak A.R., Herceg Z., Šubarić D., Babić J., Brnčić M., Brnčić S.R., Bosiljkov T., Čvek D., Tripalo B., Gelo J. (2010). Ultrasound effect on physical properties of corn starch. Carbohydr. Polym..

[39] Jiang S., Ding J., Andrade J., Rababah T.M., Almajwal A., Abulmeaty M.M., Feng H. (2017). Modifying the physicochemical properties of pea protein by pH-shifting and ultrasound combined treatments. Ultrason. Sonochemistry.

[40] Salvia-Trujillo L., Rojas-Graü M.A., Soliva-Fortuny R., Martín-Belloso O. (2013). Effect of processing parameters on physicochemical characteristics of microfluidized lemongrass essential oil-alginate nanoemulsions. Food Hydrocoll..

[41] Lin L., Gu Y., Cui H. (2019). Moringa oil/chitosan nanoparticles embedded gelatin nanofibers for food packaging against Listeria monocytogenes and Staphylococcus aureus on cheese. Food Packag. Shelf Life.

[42] Acevedo-Fani A., Salvia-Trujillo L., Rojas-Graü M.A., Martín-Belloso O. (2015). Edible films from essential-oil-loaded nanoemulsions: Physicochemical characterization and antimicrobial properties. Food Hydrocoll..

[43] Prakash A., Baskaran R., Vadivel V. (2020). Citral nanoemulsion incorporated edible coating to extend the shelf life of fresh cut pineapples. Lwt.

[44] Lu F., Liu D., Ye X., Wei Y., Liu F. (2009). Alginate–calcium coating incorporating nisin and EDTA maintains the quality of fresh northern snakehead (*Channa argus*) fillets stored at 4 C. J. Sci. Food Agric..

[45] Huang J., Chen Q., Qiu M., Li S. (2012). Chitosan-based edible coatings for quality preservation of postharvest whiteleg shrimp (*Litopenaeus vannamei*). J. Food Sci..

[46] Nirmal N.P., Benjakul S. (2011). Retardation of quality changes of Pacific white shrimp by green tea extract treatment and modified atmosphere packaging during refrigerated storage. Int. J. Food Microbiol..

[47] Shamshad S., Riaz M., Zuberi R., Qadri R. (1990). Shelf life of shrimp (*Penaeus merguiensis*) stored at different temperatures. J. Food Sci..

[48] Maqsood S., Benjakul S., Shahidi F. (2013). Emerging role of phenolic compounds as natural food additives in fish and fish products. Crit. Rev. Food Sci. Nutr..

[49] Bajpai V., Rahman A., Dung N., Huh M., Kang S. (2008). In vitro inhibition of food spoilage and foodborne pathogenic bacteria by essential oil and leaf extracts of Magnolia liliflora Desr. J. Food Sci..

[50] Benjakul S., Visessanguan W., Tanaka M. (2005). Properties of phenoloxidase isolated from the cephalothorax of kuruma prawn (*Penaeus japonicus*). J. Food Biochem..

[51] Jang M.S., Sanada A., Ushio H., Tanaka M., Ohshima T. (2003). Inhibitory effect of enokitake extract on melanosis of shrimp. Fish. Sci..

[52] Balti R., Mansour M.B., Zayoud N., Le Balc’h R., Brodu N., Arhaliass A., Masse A. (2020). Active exopolysaccharides based edible coatings enriched with red seaweed (*Gracilaria gracilis*) extract to improve shrimp preservation during refrigerated storage. Food Biosci..

[53] Wu G., Farouk M., Clerens S., Rosenvold K. (2014). Effect of beef ultimate pH and large structural protein changes with aging on meat tenderness. Meat Sci..

[54] Morachis-Valdez A.G., Gómez-Oliván L.M., García-Argueta I., Hernández-Navarro M.D., Díaz-Bandera D., Dublán-García O. (2017). Effect of chitosan edible coating on the biochemical and physical characteristics of carp fillet (*Cyprinus carpio*) stored at−18 C. Int. J. Food Sci..

[55] Soyer A., Özalp B., Dalmış Ü., Bilgin V. (2010). Effects of freezing temperature and duration of frozen storage on lipid and protein oxidation in chicken meat. Food Chem..

[56] Hematyar N., Rustad T., Sampels S., Kastrup Dalsgaard T. (2019). Relationship between lipid and protein oxidation in fish. Aquac. Res..

[57] Bazargani-Gilani B., Aliakbarlu J., Tajik H. (2015). Effect of pomegranate juice dipping and chitosan coating enriched with Zataria multiflora Boiss essential oil on the shelf-life of chicken meat during refrigerated storage. Innov. Food Sci. Emerg. Technol..

[58] Ansarian E., Aminzare M., Azar H.H., Mehrasbi M.R., Bimakr M. (2022). Nanoemulsion-based basil seed gum edible film containing resveratrol and clove essential oil: In vitro antioxidant properties and its effect on oxidative stability and sensory characteristic of camel meat during refrigeration storage. Meat Sci..

[59] Rysman T., Van Hecke T., Van Poucke C., De Smet S., Van Royen G. (2016). Protein oxidation and proteolysis during storage and in vitro digestion of pork and beef patties. Food Chem..

[60] Lorenzo J.M., Gómez M. (2012). Shelf life of fresh foal meat under MAP, overwrap and vacuum packaging conditions. Meat Sci..

[61] Rajasekaran B., Singh A., Nagarajan M., Benjakul S. (2022). Effect of chitooligosaccharide and α-tocopherol on physical properties and oxidative stability of shrimp oil-in-water emulsion stabilized by bovine serum albumin-chitosan complex. Food Control.

[62] Reesha K., Panda S.K., Bindu J., Varghese T. (2015). Development and characterization of an LDPE/chitosan composite antimicrobial film for chilled fish storage. Int. J. Biol. Macromol..

[63] Medina I., Gallardo J., González M.J., Lois S., Hedges N. (2007). Effect of molecular structure of phenolic families as hydroxycinnamic acids and catechins on their antioxidant effectiveness in minced fish muscle. J. Agric. Food Chem..

[64] Souza J.N., Silva E.M., Loir A., Rees J.-F., Rogez H., Larondelle Y. (2008). Antioxidant capacity of four polyphenol-rich Amazonian plant extracts: A correlation study using chemical and biological in vitro assays. Food Chem..

[65] Dehghani S., Hosseini S.V., Regenstein J.M. (2018). Edible films and coatings in seafood preservation: A review. Food Chem..

[66] Buamard N., Benjakul S. (2017). Ethanolic coconut husk extract: In vitro antioxidative activity and effect on oxidative stability of shrimp oil emulsion. Eur. J. Lipid Sci. Technol..

[67] Xu D., Xue H., Sun L., Wang Y. (2018). Retardation of melanosis development and quality degradation of Litopenaeus vannamei with starving treatment during cold storage. Food Control.

[68] Shahidi F., Wanasundara P., Akoh C.C., dan Min D.B. (2002). Extraction and analysis of lipids. Food Lipids, Chemistry Nutrition and Biotechnology.

[69] Chi S., Zivanovic S., Penfield M. (2006). Application of chitosan films enriched with oregano essential oil on bologna–active compounds and sensory attributes. Food Sci. Technol. Int..

[70] Arfat Y.A., Benjakul S., Vongkamjan K., Sumpavapol P., Yarnpakdee S. (2015). Shelf-life extension of refrigerated sea bass slices wrapped with fish protein isolate/fish skin gelatin-ZnO nanocomposite film incorporated with basil leaf essential oil. J. Food Sci. Technol..

[71] Castro P., Millan R., Pennedo J.C., Sanjuan E., Santana A., Caballero M.J. (2012). Effect of storage conditions on total volatile base nitrogen determinations in fish muscle extracts. J. Aquat. Food Pro. Technol..

[72] European Commission The European Commission 1995–2000. https://op.europa.eu/en/publication-detail/-/publication/53418368-63b0-456a-95a5-c704de93a5f9/language-en.

[73] Wu T.H., Bechtel P.J. (2008). Ammonia, dimethylamine, trimethylamine, and trimethylamine oxide from raw and processed Fish by-products. J. Aquat. Food Prod. Technol..

[74] Olatunde O.O., Benjakul S., Vongkamjan K. (2019). Combined effect of ethanolic eoconut husk extract and modified atmospheric packaging (MAP) in extending the shelf life of Asian sea bass slices. J. Aquat. Food Prod. Technol..

[75] Alparslan Y., Metin C., Yapici H.H., Baygar T., Gunlu A., Baygar T. (2017). Combined effect of orange peel essential oil and gelatin coating on the quality and shelf life of shrimps. J. Food. Saf. Food Qual..

[76] Mohebi E., Shahbazi Y. (2017). Application of chitosan and gelatin based active packaging films for peeled shrimp preservation: A novel functional wrapping design. LWT-Food Sci. Technol..

[77] Liu X., Jia Y., Hu Y., Xia X., Li Y., Zhou J., Liu Y. (2016). Effect of Citrus wilsonii Tanaka extract combined with alginate-calcium coating on quality maintenance of white shrimps (*Litopenaeus vannamei* Boone). Food Control.

[78] Ragasa C.Y., Labrador P., Rideout J.A. (2006). Antimicrobial terpenoids from Lansium domesticum. Philipp. Agric. Sci..

[79] Munir T., Munawar K.S., Mohyuddin A. (2018). An overview of the antibacterial implications of Lansium domesticum. J. Basic Appl. Sci..

[80] Choulitoudi E., Ganiari S., Tsironi T., Ntzimani A., Tsimogiannis D., Taoukis P., Oreopoulou V. (2017). Edible coating enriched with rosemary extracts to enhance oxidative and microbial stability of smoked eel fillets. Food Packag. Shelf Life.

[81] Sallam K.I. (2007). Antimicrobial and antioxidant effects of sodium acetate, sodium lactate, and sodium citrate in refrigerated sliced salmon. Food Control.

[82] Alsaggaf M.S., Moussa S.H., Tayel A.A. (2017). Application of fungal chitosan incorporated with pomegranate peel extract as edible coating for microbiological, chemical and sensorial quality enhancement of Nile tilapia fillets. Int. J. Biol. Macromol..

[83] Raeisi M., Tajik H., Aliakbarlu J., Mirhosseini S.H., Hosseini S.M.H. (2015). Effect of carboxymethyl cellulose-based coatings incorporated with Zataria multiflora Boiss. essential oil and grape seed extract on the shelf life of rainbow trout fillets. LWT-Food Sci. Technol..

[84] Tsai G., Su W.-H., Chen H.-C., Pan C.-L. (2002). Antimicrobial activity of shrimp chitin and chitosan from different treatments. Fish. Sci..

